# Assessment of a novel continuous cleaning device using metatranscriptomics in diverse hospital environments

**DOI:** 10.3389/fmedt.2023.1015507

**Published:** 2023-03-03

**Authors:** Justin R Wright, Truc T Ly, Karen B Cromwell, Colin J Brislawn, Jeremy R Chen See, Samantha LC Anderson, Jordan Pellegrino, Logan Peachey, Christine Y Walls, Charise M Lloyd, Olcay Y Jones, Matthew W Lawrence, Jessica A Bess, Arthur C Wall, Alexander J Shope, Regina Lamendella

**Affiliations:** ^1^ Contamination Source Identification, LLC., Huntingdon, PA, United States; ^2^ Walter Reed National Military Medical Center, Bethesda, MD, United States; ^3^ AIONX, Hershey, PA, United States; ^4^ Nextflex, San Jose, CA, United States

**Keywords:** continuous cleaning, emergency department, oncology ward, HAI, metatranscriptomics

## Abstract

**Introduction:**

Despite routine implementation of cleaning and disinfection practices in clinical healthcare settings, high-touch environmental surfaces and contaminated equipment often serve as reservoirs for the transmission of pathogens associated with healthcare-associated infections (HAIs).

**Methods:**

The current study involved the analysis of high-touch surface swabs using a metatranscriptomic sequencing workflow (CSI-Dx™) to assess the efficacy of cleanSURFACES® technology in decreasing microbial burden by limiting re-contamination. This is a non-human single center study conducted in the Emergency Department (ED) and on an inpatient Oncology Ward of Walter Reed National Military Medical Center that have followed hygienic practices during the COVID-19 pandemic environment.

**Results:**

Although there was no difference in observed microbial richness (two-tailed Wilcoxon test with Holm correction, P > 0.05), beta diversity findings identified shifts in microbial community structure between surfaces from baseline and post-intervention timepoints (Day 1, Day 7, Day 14, and Day 28). Biomarker and regression analyses identified significant reductions in annotated transcripts for various clinically relevant microorganisms' post-intervention, coagulase-negative staphylococci and *Malassezia restricta*, at ED and Oncology ward, respectively. Additionally, post-intervention samples predominantly consisted of Proteobacteria and to a lesser extent skin commensals and endogenous environmental microorganisms in both departments.

**Discussion:**

Findings support the value of cleanSURFACES®, when coupled with routine disinfection practices, to effectively impact on the composition of active microbial communities found on high-touch surfaces in two different patient care areas of the hospital (one outpatient and one inpatient) with unique demands and patient-centered practices.

## Introduction

Healthcare-associated infections (HAIs), i.e., infections while receiving care at a healthcare facility, remain as one of the most important public health challenges for affecting 1 in 25 United States. hospital patients (CDC, 2016). In clinical settings, high-touch environmental surfaces and contaminated equipment serve as common reservoirs for the pathogens found in HAIs (1, 2). In fact, between 20% to 40% of HAIs have been estimated to be attributed to transmission *via* the hands of healthcare workers (3, 4). In the context of emergency departments (ED), these settings are characteristically fast-paced, encounter high-volumes of patients, and ultimately yield rapid patient turnover. This specific type of healthcare setting is also dynamic in which patients arrive seeking medical evaluation and treatment for a wide variety of acute illnesses or injuries. With roughly 130 million visits in 2018, EDs are the gateway for admission to inpatient medical care in the United States (5). These patients have the potential to transmit a broad array of communicable diseases to both healthcare workers and other patients who may be vulnerable to HAIs (6, 7). In contrast, the oncology ward primarily treats cancer patients who are at an elevated risk of contracting HAIs compared to non-cancer patients for a number of reasons including, compromised immune systems, invasive devices (i.e., catheters), and invasive surgeries (8). Both clinical settings bring unique sets of challenges for infection control.

There is a wide variety of hospital practices ranging from chemical-based disinfectants to ultraviolet light, commonly used for environmental safety. Although these methods are effective, they do not provide continuous efficacy, as the literature demonstrates surface re-contaminated occurs within minutes of decontamination (9, 10). Re-contamination of indoor surfaces is thought to occur for several reasons, including the fact that building inhabitants, like patients and caregivers, are estimated to constantly shed up to 37 million microbes/hour (11, 12). In response to the apparent need for continuous disinfection in the healthcare industry, efficacy studies on copper-alloy surfaces have shown potential in persistently reducing hospital surface microbial burden during caregiving hours (13).

More recently, AIONX® has developed a unique technology, cleanSURFACES®, that also harnesses the antimicrobial properties of copper and silver ions. Micro-electric currents that run throughout cleanSURFACES® are closed when objects like pathogens land on the product. Once the micro-electric currents are complete, concentrations of silver and copper ions are released to create a micro-environment that is toxic to pathogens. We have previously shown within an intensive care unit, that installation of cleanSURFACES® at high-touch surfaces were effective in reducing microbial burden, including *Staphylococcus* spp (13). In that study, the microbial biodiversity was assessed using a metatranscriptomic (MT) sequencing pipeline (CSI-Dx™) that allows simultaneous assessment of a large breadth of microbial diversity from a given sample, like surface swabs (14–16, 13). In various clinical scenarios, MT has shown promise in identifying active pathogens and associated pathways of interest (17, 18). We now present our recent investigation to assess and compare the efficacy of cleanSURFACES® on high-touch surfaces over time at an emergency department and oncology ward, representing two unique clinical environments which differ in risk factors for HAIs.

## Methods

### Site information, study design, and sampling methodology

This study was conducted at Walter Reed National Military Medical Center, Bethesda. A total of 91 samples were collected from the Emergency Department and Oncology ward at Walter Reed National Military Medical Center (Bethesda, MD) across two high-touch surface types (keyboards and work surfaces). Four baseline samples were collected from each of the two surface types across the two departments for a total of 16 baseline samples. cleanSURFACES® mats were then installed either atop or near surfaces. For surfaces that are not flat, like keyboards, cleanSURFACES® were installed on a nearby surface. To reduce sampling bias through the physical removal of bioburden from surfaces (19), mats were split into quadrants for sampling at each of the 4 timepoints throughout the duration of the intervention. At least eight samples were collected from surfaces at each timepoint (Day 1, Day 7, Day 14, and Day 28) after the installation of cleanSURFACES®. Findings from disinfection literature have reported surface recontamination within minutes to days after decontamination events (9, 10). Post-intervention timepoints for the current study were selected based on disinfection literature and previous cleanSURFACES® studies that demonstrated significant impacts on bioburden after two weeks (13, 20). Samples were collected following 6 h after daily disinfection throughout the duration of the study. Routine daily disinfection by environmental services were performed based on CDC protocols for floors, trash, and high-touch surfaces with diluted 3M Disinfectant Cleaner RCT concentrate (21). As per disinfection protocol at Walter Reed National Military Medical Center, nursing staff disinfected workspaces throughout the duration of their shift, in addition to staff shift changes. Nursing staff used a range of chemical-based disinfectant wipes from Professional Disposables International, Inc and Clorox. Each swab was collected using the same sampling methodology as described in Chen See et al. (13) and was immediately preserved in 2 ml of Shield DNA/RNA.

### RNA extraction, concentration, quantification, library preparation, and sequencing

RNA extraction, concentration, as well as DNA and RNA quantification of samples were conducted as described (13), with the exception of additional extraction positive controls in the current study. A volume of 1 ml of preserved *Aliivibrio fischeri* at a concentration of 1 × 10^6^ cells/ml was used for each RNA extraction positive control. Concentrated RNA extracts were normalized to 500 pg as an input for the NEBNext Single Cell/Low Input RNA Library Prep Kit (New England Biolabs, Ipswich MA). In situations where the concentration of an RNA extract was below detection (<0.25 ng/ml) the maximum volume (8 µl) was used for cDNA synthesis. Libraries were prepared based on the manufacturer's protocol for the NEBNext Single Cell/Low Input RNA Library Prep Kit. In accordance with the manufacturer's instructions, sample libraries were quantified using a Quant-iT 1 × dsDNA High Sensitivity Assay (Thermo Fisher Scientific, Waltham MA). Sequencing libraries were prepared by pooling sample libraries at an equivalent mass. Ampure XP beads (0.9 X ratio) (Beckman Coulter, Brea, CA) were then used to purify sequencing libraries. After purified sequencing libraries were quantified using a Qubit 1 × dsDNA HS assay (Thermo Fisher Scientific, Waltham MA), libraries were diluted, denatured, and sequenced on an Illumina NextSeq 550 using a 150 cycle High Output v2.5 kit.

### Bioinformatics methods and analysis

Genomics sequencing data was processed as described in Chen See et al. (13), using CSI's *Rapid Active Pathogen Identification and Detection (RAPID-Dx*®) pipeline. Normalization was performed by scaling observed taxa counts proportionally to total microbial reads per sample. A noise filter was applied on a per-sample basis, omitting taxa with both a normalized mean count < 10 and also a standard deviation greater than the mean. Alpha diversity, beta diversity, and Linear discriminant analysis of Effect Size (LEfSe) were calculated as previously described (13). The Binary Jaccard distances between samples were visualized as a network, and the degree and eccentricity of each node was calculated using the tidygraph package (22). Partitioning of binary Jaccard distance into nestedness and turnover was performed again, reporting the distance between baseline samples and subsequent timepoints. A two-tailed Wilcoxon test with Holm correction was used throughout to compare means between groups and relative to baseline, with alpha = 0.05. The association between selected taxa and sampling day during intervention was characterized using Spearman's rank correlation, followed by a t-test with the Holm correction applied.

## Results

A total of 344,957,930 million raw sequences were generated from 108 samples with 91 samples yielding adequate data across five timepoints (baseline, Day 1, Day 7, Day 14, and Day 28), two surfaces (work surface and keyboard), and two facilities (Emergency Department and Oncology ward). Refer to Table 1 for a summary of samples that were included in downstream bioinformatic analysis after quality filtration.

**Table 1:** Summary of samples that were included in downstream bioinformatic analysis after quality filtration.

** **		Timepoint				
Department	Surface Type	Baseline	AIONX®, Day 1	AIONX®, Day 7	AIONX®, Day 14	AIONX®, Day 28
**Emergency**	Keyboard	3	4	6	6	6
	Work Surface	4	4	6	6	6
**Oncology**	Keyboard	4	4	4	4	4
	Work Surface	4	4	4	4	4

### Alpha diversity

**Figure 1:**
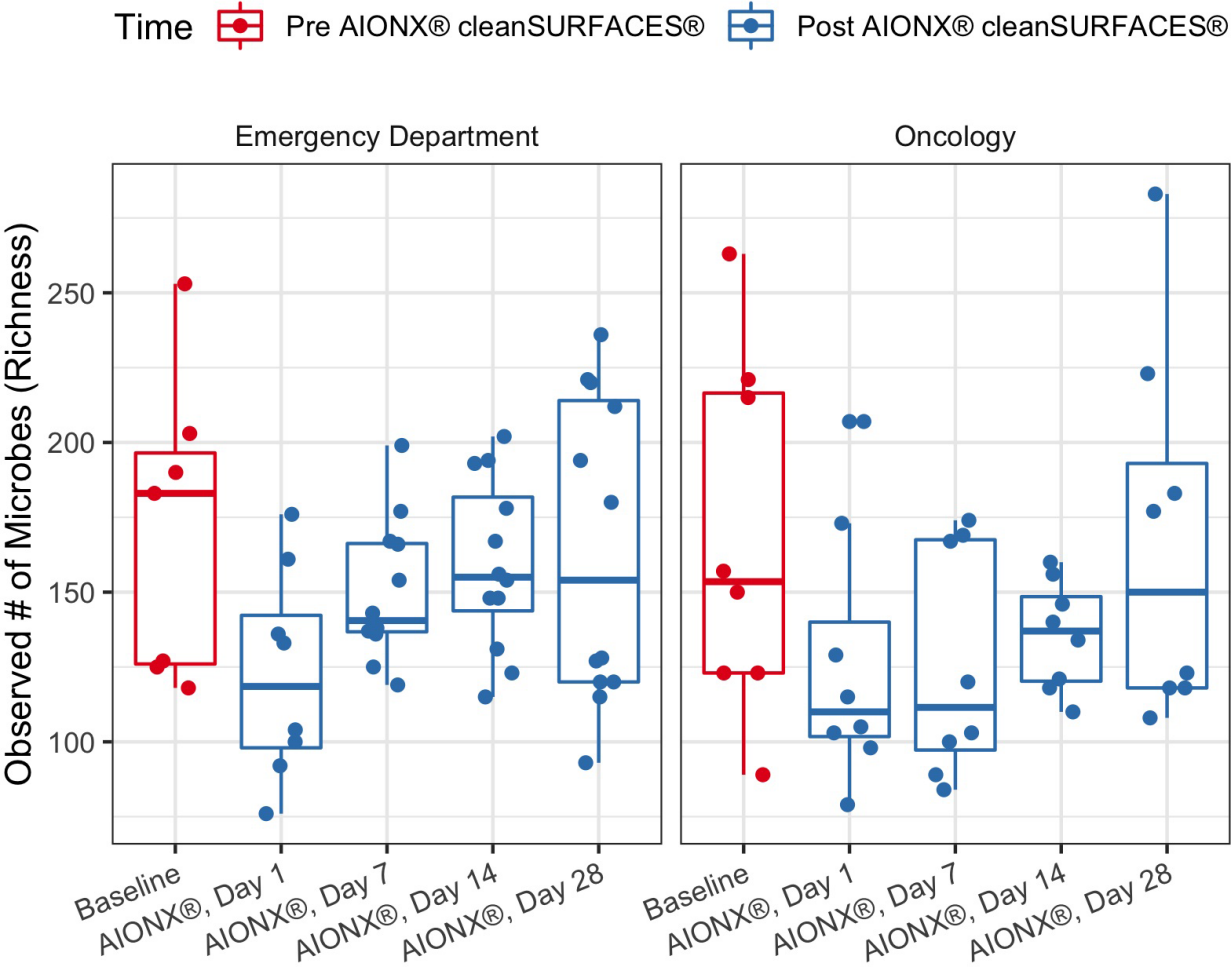
Observed microbial richness for emergency and oncology ward before (baseline) and after AIONX® intervention (Day 1, Day 7, Day 14, and Day 28). While not statistically significant, there was an observed decrease in microbial richness from baseline to Day 1 and 7 samples.

The richness of each sample was reported as the number of unique microbial taxa observed after quality filtration and was delineated by facility and timepoint post-intervention. Median microbial richness was lower after 1 Day of intervention by AIONX® compared to baseline (dropping from a median of 171 to 122 in Emergency Department and 168 to 126 in Oncology ward), and slowly returns to a similar level (164 and 167, respectively) by Day 28 (Figure 1). Across all samples similar trends were observed, with mean Richness beginning at 169, dropping to 124 by day 1, and returning to 165 on Day 28. However, variability in richness was high across all timepoints and a two-tailed Wilcoxon test followed with Holm correction showed that no timepoint was significantly different in richness compared to baseline in either facility or by surface type (Figure 1, Supplementary Figure S1).

### Beta diversity network

**Figure 2:**
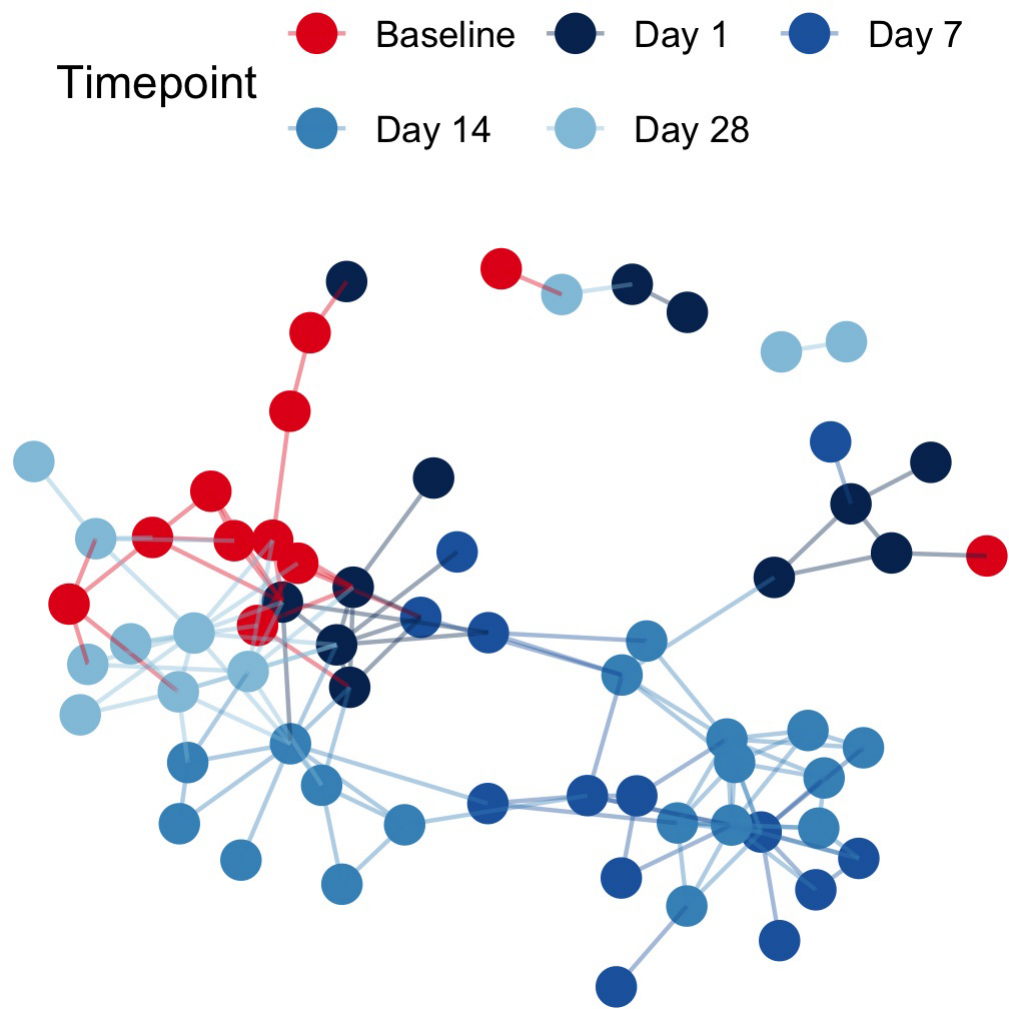
Sample networks of surface swabs connect samples with similar microbial community compositions (weighted jaccard distance < 0.4) when colored by pre/post AIONX® intervention and sampling day reveal successional changes in the microbial community over time. A total of 26 isolated vertices represented samples with < 60% taxa in common with any other samples have been omitted from the network.

Pairwise binary Jaccard distances between all samples were calculated and visualized as a network. Connected nodes represent samples that share 60% of taxa in common (binary Jaccard distance < 0.4). A total of 26 isolated nodes were omitted from the graph, while connected components were colored by pre/post cleanSURFACES® intervention and timepoint (Figure 2). Baseline samples had a median of 3 edges connecting them to other samples, while post-intervention samples, including those from Day 14, yielded a median of 4 connections between samples. A greater degree of connectivity indicates a greater number of samples that share more microbial content within a given group of samples. In the network of all samples, median node disconnectedness was higher post-intervention, peaking on Day 7. Disconnectedness measures the maximum shortest path from each sample to all other samples in the network, and the increased values on Day 7 are indicative of disruption to the sample composition during this time.

### Nestedness and turnover

**Figure 3:**
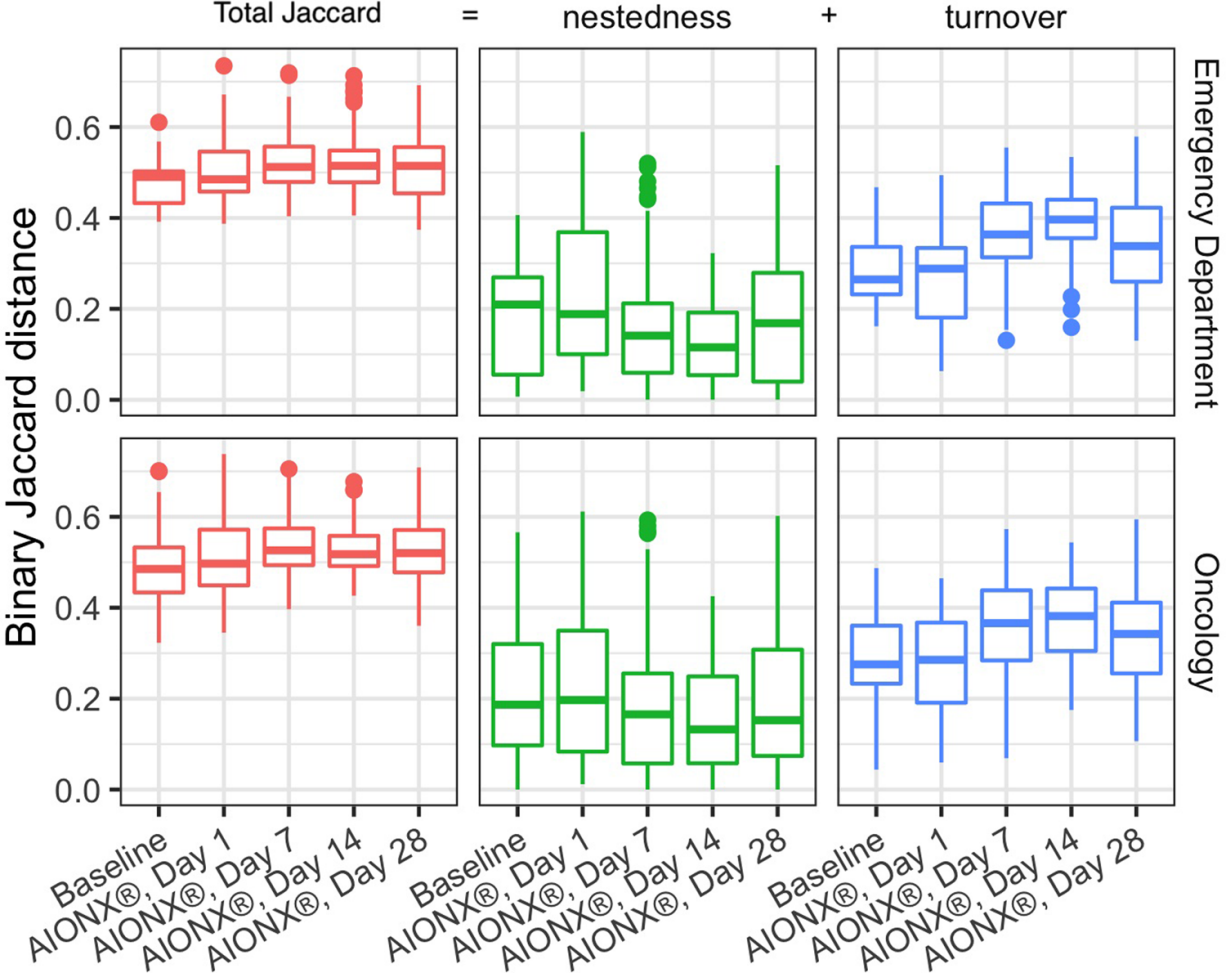
Comparisons of baseline samples to post AIONX® intervention across emergency department and oncology ward. Binary Jaccard distances were partitioned into nestedness (species loss) and turnover (species gain) to explain their contribution to changes compared to baseline. Higher Jaccard distances indicate more dissimilarity in the microbial community where as lower Jaccard distances indicate more similarity in the microbial community. Turnover contributes more to Jaccard distances as compared to nestedness. Nestedness species loss stays relatively stable over time while turnover (species gain) increases over time.

**Figure 4:**
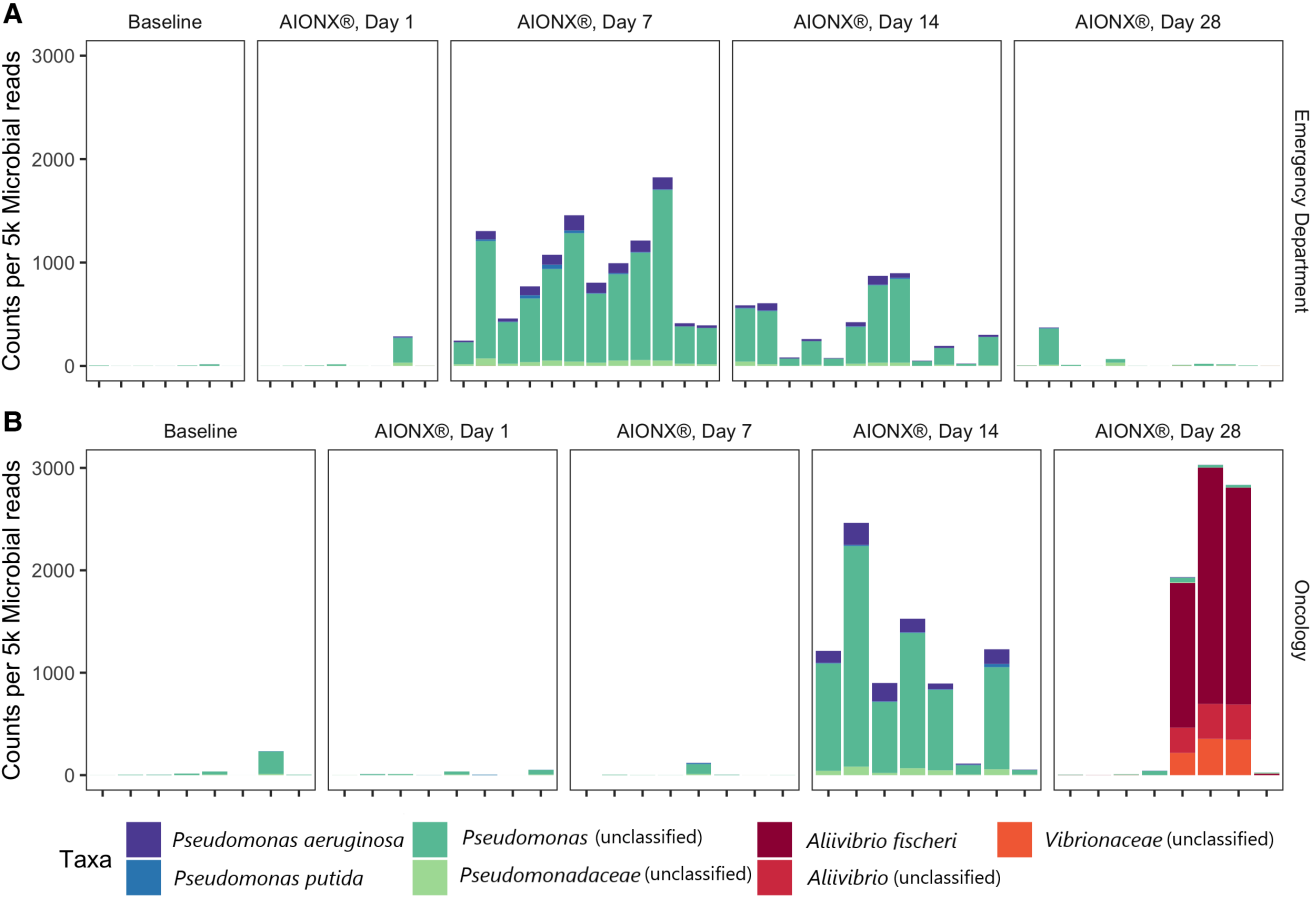
Proteobacteria from the orders *Pseudomonadales* and *vibrionales* vary over time in the emergency department (**A**) and oncology ward (**B**). Counts are normalized to 5,000 microbial reads per sample and are colored by their most specific taxonomic classification.

Samples before and after intervention were compared using the binary Jaccard distances as described above. Because any difference between a pair of samples can be attributed to a combination of nestedness (species loss) and turnover (species gain), we can partition these distances into their nestedness and turnover components to describe how the successional changes in community composition compared to baseline. For both Emergency Department and Oncology ward, median Jaccard distances increase over time from baseline to post-intervention indicating a change in the microbial community structure driven by increases in microbial turnover (Figure 3). Species gain was significantly greater than species loss across both buildings and surface types over time, which is consistent with the patterns of successional changes we observed in composition of Proteobacteria (Figure 4). Changes due to turnover in subsequent samples were also significantly greater across both the Emergency Department and Oncology ward samples on Day 7 and Day 14 after intervention (two-tailed Wilcoxon test with Holm correction, all *p* < 0.002). By contrast, nestedness was only significantly different from baseline in one group; Oncology samples from Day 14, when the median species loss compared to baseline fell under 15% (Figure 3).

### Lefse biomarker analysis

**Figure 5:**
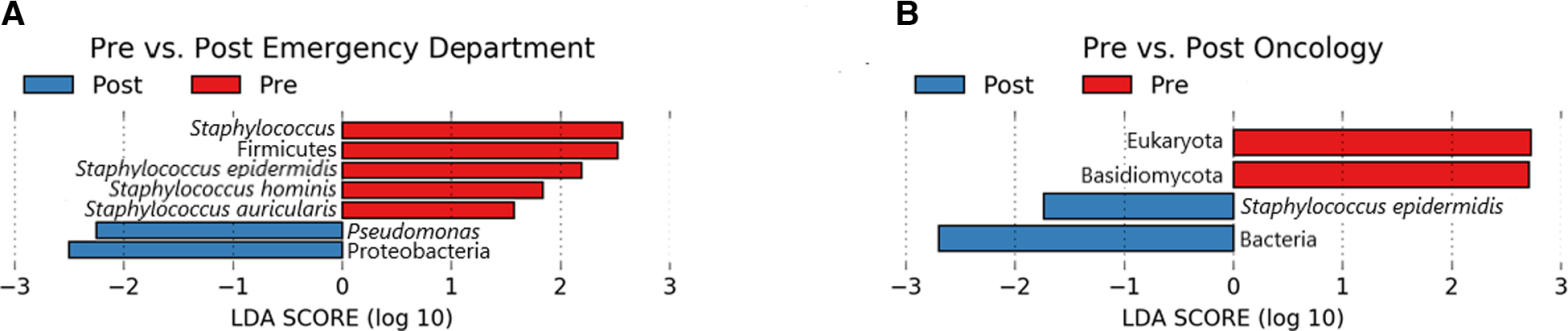
Lefse pre/post for emergency department (**A**) and oncology ward (**B**). Taxa that were enriched in Pre/baseline are indicated with red bars, while taxa that were enriched in the post-intervention samples are indicated with blue bars. Significant (Kruskal-Wallis, *p* ≤ 0.05 and log (LDA) ≥ 1.5) features are shown.

A total of 7 microbial taxa (domains, phyla, genera, and species) were significantly enriched (Kruskal-Wallis *p* ≤ 0.05, log (LDA) ≥ 1.5) among Emergency Department baseline samples compared to post-intervention samples (Figure 5). Taxa identified to be elevated in baseline samples from the Emergency Department included *Firmicutes*, *Staphylococcus*, *S. epidermidis*, *S. hominis*, and *S. auricularis* (Kruskal-Wallis *p* ≤ 0.05, log (LDA) ≥ 1.5). Within the same comparison, *Pseudomonas* and *Proteobacteria* were found to be significantly enriched in post-intervention samples. Additional biomarker analysis comparing baseline and post-intervention timepoints (Day 1, Day 7, Day 14, Day 28) revealed similar taxa to be enriched (Kruskal-Wallis *p* ≤ 0.05, log (LDA) ≥ 1.5) from baseline Emergency Department samples, in addition to *Corynebacterium* spp., *Lactobacillus*, *Clostridium*, *Leptotrichia* spp., and *Malassezia* spp. (Supplementary Figure S2). In addition to *Proteobacteria* and *Pseudomonas* previously observed (Figure 5), *P. aeruginosa*, *Stenotrophomonas*, *Leptorichia*, *Fusobacteria*, and *Actinobacteria* were significantly elevated (Kruskal-Wallis *p* ≤ 0.05, log (LDA) ≥ 1.5) at various post-intervention timepoints compared to baseline (Supplementary Figure S2).

Biomarker analysis comparing baseline and post-intervention Oncology ward samples revealed Eukaryota and *Basidiomycota* to be significantly enriched (Kruskal-Wallis *p* ≤ 0.05, log (LDA) ≥ 1.5) in baseline samples, whereas Bacteria and *S. epidermidis* was elevated among post-intervention samples (Figure 5). Additional analysis comparing baseline to each of the post-intervention timepoints also revealed *Basidiomycota*, in addition to *Lactobacillus curvatus*, *Streptococcus salivarious*, and *M. restricta* to be enriched (Kruskal-Wallis *p* ≤ 0.05, log (LDA) ≥ 1.5) in baseline Oncology ward samples (Supplementary Figure S3). For the baseline-Day 1 and baseline-Day 7 comparison only *S. epidermidis* was significantly elevated in the post-intervention timepoint. Two comparisons of Oncology ward samples, baseline-Day 14 and baseline-Day 28, yielded different significantly enriched taxa that included *P. aeuriginosa, P. fluorescens*, *Stenophomonas*, *Tobamovirus*, *Cutibacterium acnes*, and *Aliivibrio fischeri* (Supplementary Figure S3). Overall, biomarker analysis comparing baseline samples to post-intervention samples separated based on surface yielded additional active microbial taxa within both the Emergency Department and Oncology ward.

### Observed shifts in abundance of potential opportunistic pathogens

**Figure 6:**
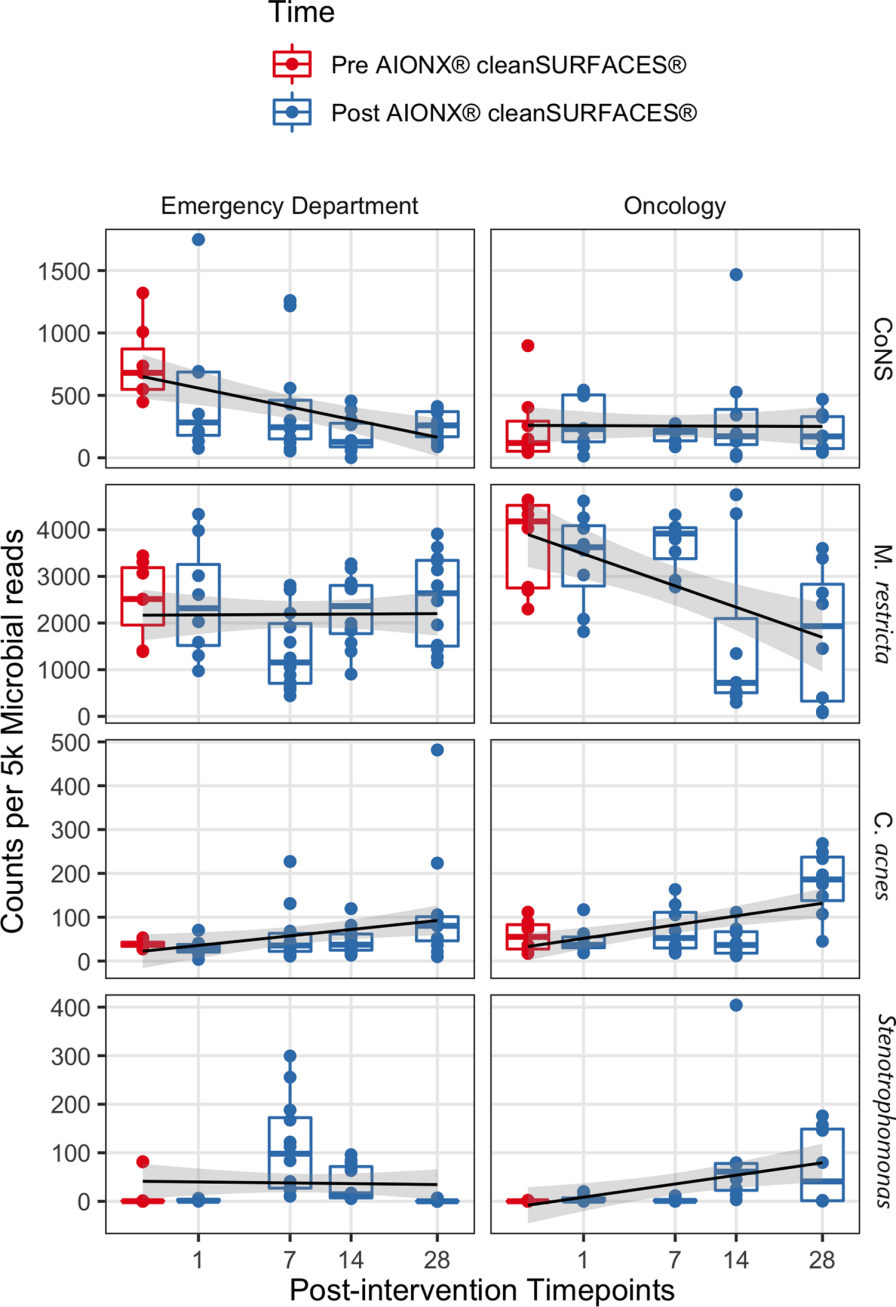
The normalized counts of clinically relevant taxa associated with HAIs are shown before and after AIONX® intervention. A linear regression shows counts per 5 k microbial reads depend on the square root of days post-intervention for each taxon and building, with a standard error of 95% shown in gray.

Coagulase-negative staphylococci (CoNS) was uncommon in the Oncology ward, with a median normalized count under 300 at all timepoints (Figure 6). However, the abundance of CoNS sharply decreased in samples from the Emergency Department and shows a negative correlation of −0.4 across all timepoints (*p* = 0.02). Normalized counts of Proteobacteria increased in both departments post-intervention, particularly on Day 7 in the Emergency Department and Day 14 in the Oncology ward (Figure 4), where the median counts of Pseudomonas increased to 750 and 893 respectively. The increased abundance of Proteobacteria in Day 28 from the Oncology ward is attributed to barcode hopping from a deeply sequenced extraction positive control containing 5.45 million reads of *A. fischeri,* given that *A. ficheri* makes up >30% of these three samples, but less than 0.5% of any other sample in the project, and is a free-living marine microbe (Figure 4). This pattern was contrasted by *Malassezia restricta*, which was consistent in the Emergency Department but showed a progressive decrease over time in Oncology ward (*p* = 0.005). Some microbes did increase in relative abundance over the course of the study: *Cutibacterium acnes* showed positive correlations to sampling day in both Emergency Department and Oncology ward (*p* > 0.05) and *Stenotrophomonas* showed a positive correlation of.6 in Oncology ward (*p* < 0.001).

## Discussion

To date there is a single study that assessed the efficacy of cleanSURFACES® in an intensive care unit (13). The current study is unique in which the cleanSURFACES® intervention was implemented in two distinct departments (Emergency Department and Oncology ward) at the Walter Reed National Military Medical Center. Emergency departments are a distinct clinical setting in which patients and healthcare workers are potentially exposed to a large burden of communicable diseases, since these departments serve as a primary point of access to healthcare (7, 6, 5). On the other hand, oncology wards care for immunocompromised patient who are largely subject to invasive procedures throughout the duration of treatment (8). The clinical and economic burden of HAIs have been extensively assessed and ultimately highlight the importance of implementing effective infection prevention and disinfection policies for both types of clinical settings (23, 24). Considering the inherent vulnerabilities associated (i.e., surface recontamination) with current infection prevention and control practices, the current study sought to assess the efficacy of cleanSURFACES® technology in both the Emergency Department and Oncology ward at the Walter Reed National Military Medical Center.

### Alpha and Beta diversity findings

While observed richness decreases from baseline to early post-intervention timepoints, this trend is not statistically significant (Figure 1). Interestingly, a previous study by Chen See et al. (13) observed significant differences between baseline and post-intervention timepoints. The observed stability in microbial richness in the current study is likely attributed to rigorous disinfection protocols carried out at Walter Reed National Military Medical Center, where the nursing staff are required to disinfect surfaces throughout and between shifts, in addition to daily disinfection.

While alpha diversity is a useful index in describing the number of unique taxa present in samples across a study, beta-diversity reveals compositional comparisons within microbial communities between samples. post-intervention samples were more compositionally similar in microbial content as compared to baseline samples, suggesting a marked shift in microbial community structure post-intervention (Figure 2). In particular, elevated disconnectedness among post-intervention samples were revealed, particularly Day 7 samples, suggesting successional changes in the microbial community post-intervention. Previous surface disinfection studies have also observed successional shifts in microbial community composition (13). This is particularly attributed to the fact that associated microbiomes of indoor built environments are not static and are impacted by variations in building occupants over time since humans are estimated to shed 37 million microbes per hour (11, 12). Additionally, variations in building design/operation (i.e., HVAC systems) have also been shown to impact the composition of indoor microbial communities (25). In comparison to baseline samples, shifts to the microbial community composition of post-intervention samples, especially during Day 7 and Day 14, were attributed to turnover (species gained) (Figure 3). Although microbial composition is different between baseline and post-intervention samples (Figure 1), it should be noted that a consistent number of unique microbial taxa before and after intervention was observed. This finding suggests that the intervention in the current study maintained a steady state count of unique microorganisms.

### Diversity of microbes present on post-intervention surfaces

Proteobacteria were shown to be present during several post-intervention timepoints (Figure 4). Day 14 and 28 timepoints yielded an increased normalized counts of unclassified *Pseudomonas* and *Aliivibrio fischeri* within the Oncology ward samples respectively. In particular, annotated transcripts of A. *fischeri* were present in three of the Day 28 Oncology samples. Incidentally, this microorganism was utilized as an extraction positive control in the current study. A. *fischeri* is a bioluminescent microorganism and is native to marine environments (26); to our knowledge, this microorganism has not been isolated from indoor surfaces or clinical settings. Since the presence of A. *fischeri* on clinical surfaces are unlikely based on existing ecological studies, it is suspected that barcode hopping occurred during the sequencing process because the extraction positive control containing this microorganism was deeply sequenced (5.45 million reads). Samples from the Emergency Department were also observed to have elevated normalized counts of unclassified *Pseudomonas* post-intervention at Day 7 and 14 (Figure 4). By Day 28 in both the Emergency Department and Oncology ward, normalized counts of unclassified *Pseudomonas* returned to levels resembling baseline samples. While the reason behind these observations remain unclear, it could have been attributed to the degree of disinfection intensity and type of chemical disinfectants mats were subject to throughout the duration of the study in both sites. In a previous study that also utilized metatranscriptomics to characterize the efficacy of cleanSURFACES®, several mats were reported to be non-functional by the end of the study period due to repeated disinfection with chemical-based wipes (13). Other studies that have utilized NGS technology to characterize the diversity of microbial communities from various built environment settings (i.e., offices, homes) have reported Proteobacteria to be among the most ubiquitous Phyla present (27, 28). Interestingly, microbial succession primarily consisting of Proteobacteria have been reported after a series of disinfection processes (ultraviolet, ozonation and photocatalytic ozonation) for urban wastewater and surface water samples (29). Although more research in this area is required, it is speculated that selective advantages, like increased tolerance to oxidative stress and DNA repair mechanisms, may be contributing to the proliferation of Proteobacteria following these water disinfection processes (29). The phylum of Proteobacteria consists of a diverse array of microbes that range from human gut commensals (30) to environmental microorganisms (31). Findings from the current study suggest that the cleanSURFACES® intervention in conjunction with regular disinfection practices are not only impacting the active microbial community composition of high touch surfaces of in the Oncology ward but also the Emergency Department as well. In particular, the intervention may be providing selective pressure for an active microbial community composition that is initially dominated by Proteobacteria, which is consistent with observations reported from other studies that used sequencing technology to characterize surfaces from various built environment settings (27, 28).

In addition to Proteobacteria, additional biomarker and correlation analyses revealed a diverse set of microbes to be enriched throughout post-intervention timepoints in the Oncology ward, compared to samples from the Emergency Department (Supplementary Figures S2–S3). This ranged from endogenous environmental microbes, *Tobamovirus* and *Stenotrophomonas* (32, 33),, to more clinically relevant potential opportunistic pathogens like *S. epidermidis*, *C. acnes*, *Pseudomonas fluoresences*, and *Pseudomonas aeruginosa* (34–37). Although the genus, *Stenotrophomonas,* is generally associated with environmental microbes, the species *S. maltophila*, has recently emerged as a multi-drug resistant opportunistic pathogen especially for immunocompromised hospital patients (38). Among the list of potential opportunistic pathogens enriched throughout the post-intervention timepoints, only *Staphylococcus epidermidis* was enriched at Day 1 and Day 7 of the intervention (Supplementary Figure S3). The final two post-intervention timepoints were shown to enrich for a number of opportunistic pathogens that are commonly found in a range of environments including skin. *P. aeruginosa* and *P. fluorescens* were significantly enriched by Day 14, in addition to *Cutibacterium acnes* at Day 28 (Supplementary Figure S3). In addition to being a commensal gut microbe, *P. fluorescens* has also been isolated from environmental soil and is not generally considered pathogenic. However, several case studies have associated *P. fluorescens* with acute HAIs related to contaminated medical products or intravenous infusion equipment (36). While these potential opportunistic pathogens are shown to be present in post-intervention samples, they are also among the list of common skin commensals that are constantly being shed by building inhabitants like healthcare works and patients. Interestingly, several of these microorganisms (i.e., *Stenotrophomonas sp., P. aeruginosa* and *P. fluorescens)* have been reported to exhibit characteristics of copper-resistance (39–42). Copper resistance could have also contributed to the presence of several of these microorganisms on surfaces post-intervention. However, more work concerning functional genes are required in the future to confirm whether silver and copper resistance or other resistance mechanisms could be contributing to the presence of certain microorganisms on cleanSURFACES® product. Overall, between the two sites in this study, the Oncology ward was shown to have a more diverse set of microorganisms present post-intervention compared to the Emergency Department.

In addition to variation in building occupancy and metal resistance mechanisms, the presence of several types of skin commensal microorganisms in the Oncology ward could be associated with the average length of stay for patients and associated disinfection protocols. Humans are estimated to shed up to 37 million microbes/hour (11, 12). Since average hospital stays in oncology wards are generally longer (several days) than emergency departments (several hours) (43–45), the degree of accumulated microbes shed from patients throughout their length of a stay on surfaces can potentially serve as reservoirs for transmitting commensal skin microbes throughout the Oncology ward in the current study. The transmission of microorganisms can occur directly and indirectly through the hands of healthcare workers, high-touch surfaces, and medical devices (46–48). While this study did not assess hospital room surfaces and cleanSURFACES® were not installed in those areas, these surfaces remain a constituent of the whole Oncology ward surface network that healthcare workers and patients both interact with. This trend in presence of skin commensal microbes may have not been observed in the Emergency Department because that type of clinical setting generally experiences high patient turnover, and subsequently more thorough disinfection is able to occur in hospital rooms between patient visits.

### Reduced abundance of HAI-associated taxa post-intervention

In addition to Proteobacteria and other skin commensals*,* the abundance of several HAI-associated taxa also shifted throughout the duration of the current study. Counts of coagulase-negative Staphylococci (CoNS) from surfaces in the Emergency Department were significantly reduced from baseline to post-intervention samples (Figure 6). Similarly, biomarker analysis revealed several CoNS to be significantly enriched in baseline samples when compared to post-intervention timepoints (Supplementary Figure S2). While CoNS have traditionally been considered nonpathogenic commensal skin microorganisms, CoNS have recently emerged as common opportunistic pathogens causing HAIs and have been isolated from common medical equipment like stethoscopes in Emergency Departments (49–51). In addition to being among one of the most frequently recovered bacteria in routine clinical care settings (52), CoNS are also reported to be a common blood culture-assay contaminant (53). The clinical significance of CoNS is extensive and range from vulnerable groups like neonates and immunocompromised patients, to HAIs associated with invasive medical devices (i.e., cardiac valves, joint replacements), and bloodstream infections (54). Current literature concerning the surveillance of CoNS-associated infections highlight the importance in the ability to identify infections where CoNS are truly the causative agent of an HAI rather than a remnant of contamination (55). While most cases of blood culture assay contamination are associated with the method of specimen collection, environmental sources of contamination have also been studied (56). Results from the current study suggest that the cleanSURFACES® intervention may be contributing to the reduction of active transcripts associated with CoNS on high-touch surfaces in the Emergency Department. However, more clinically focused research is required to assess the clinical impact of cleanSURFACES® on the prevalence of CoNS-associated HAIs.

Like the observed decrease in abundance of CoNS in the Emergency Department, *M. restricta* was also reduced from baseline to post-intervention in the Oncology ward (Figure 6). Additional biomarker analysis revealed *Basidiomycota* and *M. restricta* and to be significantly enriched in baseline samples compared to Day 14 and 28 post-intervention samples (Supplementary Figure S3). In support of these findings, a previous study that sought to assess the efficacy of cleanSURFACES® in an intensive care unit found both *Basidiomycota* and *M. restricta* to be largely present in baseline samples. As one of the most ubiquitous Malassezia species, *M. restricta*, is a fungal opportunistic pathogen that has been isolated from immunocompromised oncology patients (57). The phylum, *Basidiomycota,* contains a diverse array of ubiquitous outdoor fungal species. However, in recent years, several filamentous basidiomycetes have emerged as important clinical pathogens (58). These findings suggest that the cleanSURFACES® intervention could also contribute to the reduction to various kinds of ubiquitous fungal opportunistic pathogens. However, additional research is required to determine whether cleanSURFACES® impact the prevalence of HAIs associated with fungal opportunistic pathogens, especially in clinical settings like oncology ward that provide care for immunocompromised patients.

### Limitations & challenges

Several limitations exist in relation to the study design of the experiment. Matched controls were not included throughout sampling timepoints and ultimately limited our ability to determine whether results were primarily attributed to the cleanSURFACES® intervention or other unaccounted factors. To our knowledge, metatranscriptomic references for indoor hospital environments that could have served as a control for the current study don't exist. Matched controls for the intervention were ultimately not included in the study because the presence of functional cleanSURFACES® would introduce bias into the network of surface microbial communities in each department. However, as described in the methods section, healthcare workers in both departments were instructed to not alter disinfection practices and how they would interact with surfaces throughout the study. Additionally, cleanSURFACES® technology was not installed on surfaces in patient rooms due to HIPAA compliance. Results reported in the current study could have been impacted by surfaces that were not accounted for since healthcare workers interact with a variety of surfaces in patient rooms that are not exclusively the surfaces that were sampled (central nursing workstations and keyboards). Statistical comparisons that split samples by several metadata categories (i.e., surface type, department, and timepoint) was also a point of limitation since groups contained a minimum count of 3 samples. Lastly, networks generated in the current study are dependent on an arbitrary threshold used to define connectivity between each pair of samples. While there is a range of pairwise binary Jaccard distances, only a single threshold can be used to define connectivity in the context of a single network.

## Conclusions

The current study confirms the impact of cleanSURFACES® on microbial biodiversity using a novel metatranscriptomics sequencing workflow (CSI-Dx™) when tested in a hospital setting. Similar to our previous observations in an intensive care unit, the data presented confirms targeting high touch areas by new technological adaptations will be valuable in different clinical environments: implementing cleanSURFACES® in two diverse clinical environments demonstrated impacts on trends of microbial activity on high-touch surfaces within the duration of the study. Factors that influenced the extent of cleanSURFACES® technology included, hospital site location, variations in disinfection protocols, and occupants in those areas. Future work involving cleanSURFACES® should be more clinically oriented and assess whether this technology impacts the prevalence of HAIs. Additionally, functional genes (i.e., antibiotic resistance genes) should also be considered in future studies.

## Data Availability

The datasets presented in this study can be found in online repositories. The names of the repository/repositories and accession number(s) can be found below: https://www.ncbi.nlm.nih.gov/, PRJNA803198.
