# Supplementary material for: Assessment of a novel continuous cleaning device using metatranscriptomics in diverse hospital environments
Source: Front Med Technol. 2023 Mar 3;5:1015507. doi: 10.3389/fmedt.2023.1015507 (PMC10020724; doi:10.3389/fmedt.2023.1015507)
Supplement: Supplementary file 1 [file Datasheet1.docx]

Supplementary Material


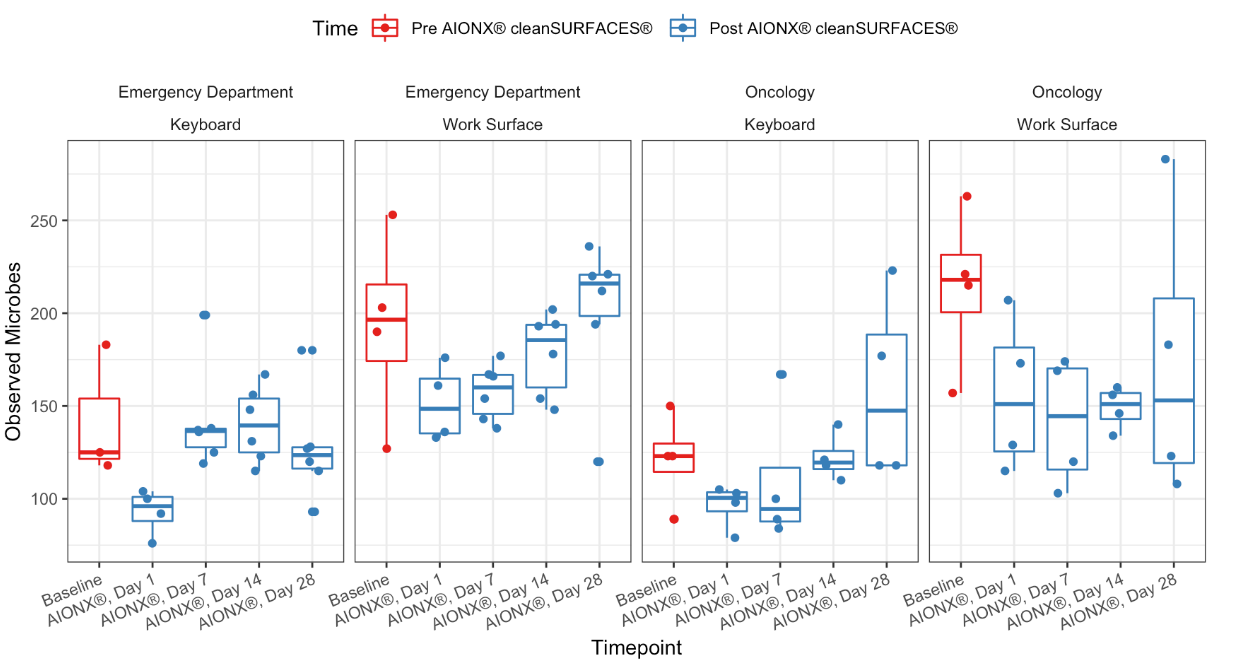


**Supplemental Figure 1.** Observed Microbial Richness during cleanSURFACES® Intervention. The number of unique taxa observed more than twice in each sample are reported. Pairwise Wilcoxon tests with a Holm correction were performed against Baseline. Observed Microbial Richness for both surface types (Keyboard, Work Surface) in the Emergency Department and Oncology Ward by timepoint (Baseline, Day 1, Day 7, Day 14, and Day 28).


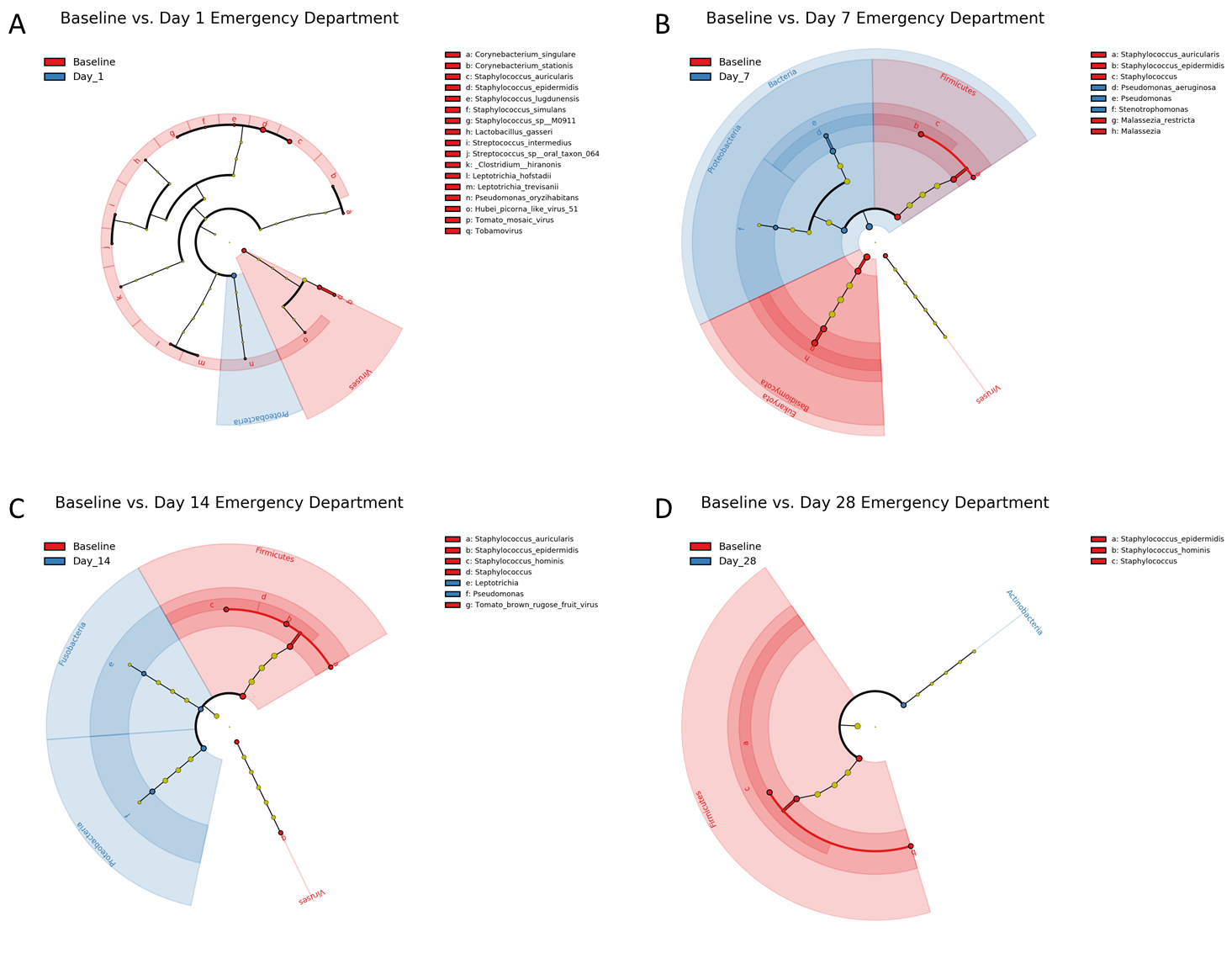


**Supplemental Figure 2.** LEfSe cladograms of comparisons between Pre/Baseline and Post-intervention timepoints: (A) Day 1, (B) Day 7, (C) Day 14, and (D) Day 28 for the Emergency Department. Taxa that were enriched in Pre/Baseline are indicated with red bars, while taxa that were enriched in the Post intervention samples are indicated with blue bars. Significant (Kruskal-Wallis, p ≤ 0.05 and log (LDA)≥1.5) features are shown.


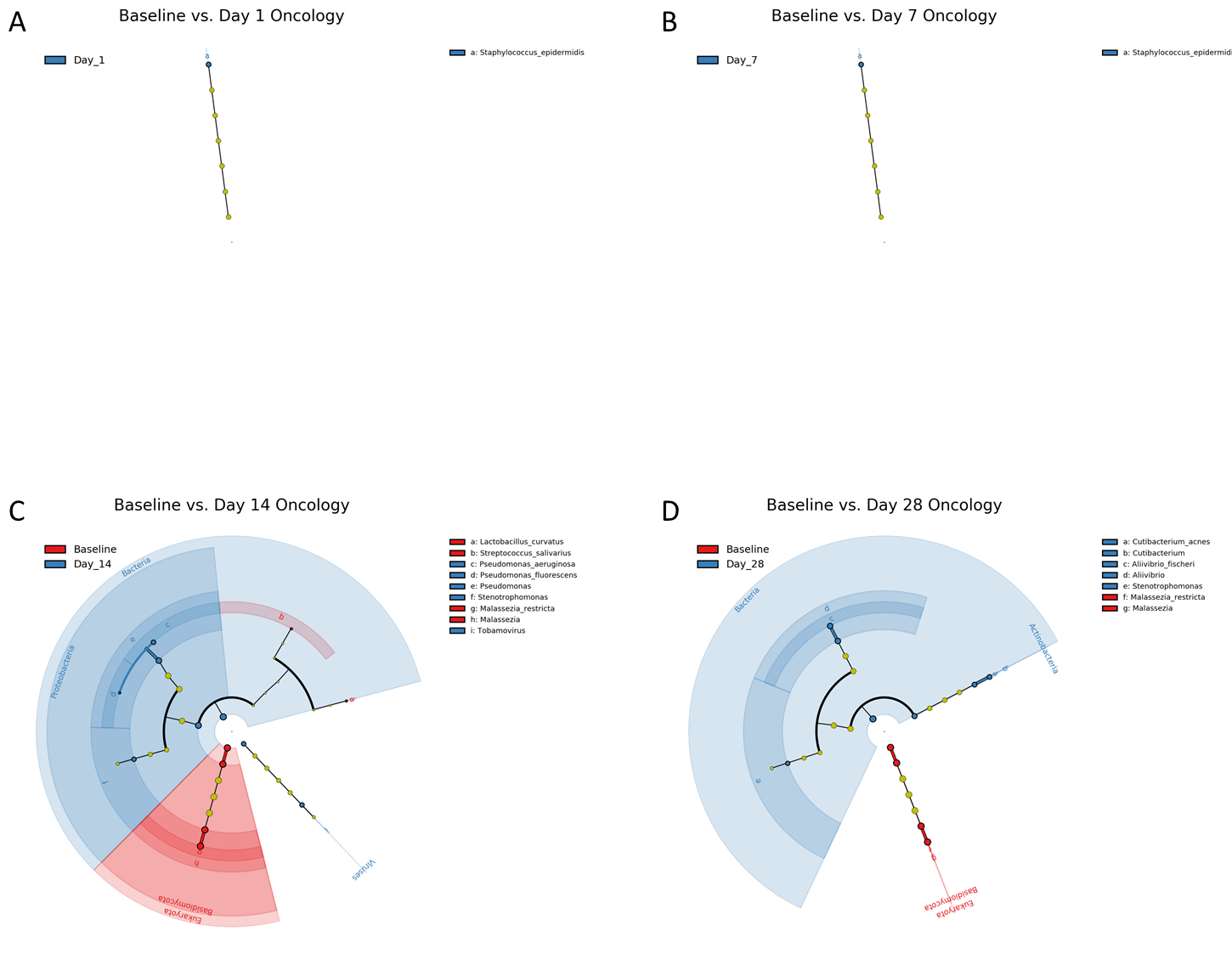


**Supplemental Figure 3.** LEfSe cladograms of comparisons between Pre/Baseline and Post-intervention timepoints: (A) Day 1, (B) Day 7, (C) Day 14, and (D) Day 28 for the Oncology Ward. Taxa that were enriched in Pre/Baseline are indicated with red bars, while taxa that were enriched in the Post intervention samples are indicated with blue bars. Significant (Kruskal-Wallis, p ≤ 0.05 and log (LDA)≥1.5) features are shown.
